# Oregano and thyme by-products of olive oil aromatization process with microwave assisted extraction as a rich source of bio-active constituents

**DOI:** 10.3389/fnut.2024.1372263

**Published:** 2024-05-09

**Authors:** Evanthia Dina, Antigoni Cheilari, Argyro Vontzalidou, Dimitra Karamani, Ioanna Diamanti, Panagiotis Bagatzounis, Ilias Giannenas, Katerina Grigoriadou, Nektarios Aligiannis

**Affiliations:** ^1^Department of Pharmacognosy and Natural Products Chemistry, Faculty of Pharmacy, National and Kapodistrian University of Athens, Athens, Greece; ^2^Pellas Nature S.A., Edessa, Greece; ^3^Bagatzounis & Sons S.A., Kozani, Greece; ^4^Laboratory of Nutrition, School of Veterinary Medicine, Thessaloniki, Greece; ^5^Institute of Plant Breeding and Genetic Resources, Hellenic Agricultural Organization (ELGO) – DIMITRA, Thessaloniki, Greece

**Keywords:** aromatization, aromatized olive oil, oregano, thyme, microwave assisted extraction, ultrasound assisted extraction, plant by-products, enriched extracts

## Abstract

**Introduction:**

Processing of Medicinal Aromatic Plants (MAPs) results in the production of a significant amount of by-products, which are not commercially exploitable. Towards this direction, we studied extensively the by-products of oregano and thyme, remaining after the aromatization of olive oils with microwave assisted extraction (MAE). The purpose of the study was the exploitation of the “wastes” of these two economically significant herbs of Greece, for the potential development of innovative bioactive products.

**Methods:**

Hence, superior and inferior quality plant material from *Origanum vulgare* subsp. *hirtum* and *Thymus vulgaris*, were extracted with extra virgin olive oil using MAE. For the evaluation of raw plant material, beside the characterization of the essential oils (EOs), the hydroalcoholic extracts of superior and inferior plant material were afforded by ultrasound assistant extraction (UAE). In addition, the remaining plant material after the flavoring of olive oil by MAE, was extracted with c-Hex, MeOH, H_2_O:MeOH using UAE. All the extracts were evaluated for their DPPH free radical scavenging activity and total phenolic content (TPC) as well as their chemical profile was investigated by HPTLC. In parallel, the EOs, the olive oils and the c-Hex extracts were analyzed by GC–MS and Headspace Solid Phase Microextraction (HS-SPME)-GC–MS.

**Results and Discussion:**

The results showed that the composition of the EOs and the volatile fraction of the olive oil extracts were similar for the superior quality material whereas for the inferior the composition of the volatile fraction of olive oil extracts was not analogous to the respective EOs. GC–MS analyses of oregano and thyme by-products revealed the presence of carvacrol, thymol, γ-terpinene and p-cymene among the major constituents. Moreover, the hydroalcoholic extracts obtained from the plant material remaining after olive oil flavoring with MAE showed similar phenolic content and scavenging activity with the hydroalcoholic extracts of the corresponding raw plant materials underlying their potent use in the preparation of high-added value products such as nutraceuticals and cosmeceuticals as well as enriched animal nutrition products.

## 1 Introduction

Olive oil is considered one of the most important element of the Mediterranean diet, well-known for its biological properties (1). Flavoring of olive oil with herbs and spices is common in Mediterranean countries since antiquity, while nowadays, different techniques (infusion, malaxation, maceration, ultrasound assisted extraction) have been employed to produce enriched aromatic oils (2–4). Beside culinary purposes, the aromatization of olive oil has been proved to increase its shelf life (thermal stability, resistance to oxidation) (4, 5) and provide unique functional food products combining the health benefits of olive oil and infused herbs (2, 6). However, the aromatization process of olive oil with medicinal and aromatic plants (MAPs), results in the production of significant amounts of by-products that have not been systematically investigated. In any case, the plant material remaining after the infusion of olive oil is considered waste and discarded. At the frame of this work, we demonstrate for the first time an in-depth study of by-products remaining after the aromatization of olive oil with oregano and thyme by microwave assisted extraction (MAE). MAE is an emerging green technique that has been employed in the extraction of natural products (7), but, so far only three times in the favoring of olive oils with plants (8–10), and the tests have been performed with domestic/modified microwave ovens. To the best of our knowledge, in this study, for the first time the analysis of volatile compounds after the infusion of olive oils with oregano (*Origanum vulgare* subsp. *hirtum* L.) and thyme (*Thymus vulgaris* L.) by MAE is presented. In this case, extra virgin olive oil from Koroneiki variety was aromatized with superior (commercially acceptable from the market) and inferior (branches/non-commercially acceptable) quality plant material and the content of volatile substances of oils was characterized by HS-SPME-GC–MS. Moreover, the plant material by-products remaining after MAE were extracted by UAE, and their chemical profile was compared by High Performance Thin Layer Chromatography (HPTLC) and/or GC–MS analyses. The extracts were evaluated for their total phenolic content (TPC) and 2,2-diphenyl-1-picrylhydrazyl (DPPH) free radical scavenging activity.

The aim of this study was not only to investigate the aromatization of olive oil with superior and inferior plant material, but also to evaluate the plant by-products remaining after MAE. The rich chemical profile of the latter provides evidence for their further valorization. The presented work is a continuation of our research on post-harvesting and processing (11, 12) by-products obtained from various raw plant materials with the purpose to highlight them as rich sources of bioactive constituents for the production of innovative products, such as animal nutrition feed additives, food supplements, cosmeceuticals and/or nutraceuticals.

## 2 Materials and methods

### 2.1 Chemicals, standards and materials

Analytical grade methanol (MeOH) and cyclohexane (c-Hex) for extraction, as well as acetonitrile (ACN), ethyl acetate (EtOAc), acetic acid (AA), sulfuric acid (H_2_SO_4_) and vanillin for HPTLC analysis, ethanol 96% (EtOH) for bioassays and potassium hydroxide (KOH) were purchased from Merck (Merck, Darmstadt, Germany). Distilled water was produced from LaboStar Pro TWF UV ultra-pure water system (Evoqua Water Technologies, Barsbuettel, Germany). For free radical scavenging and total phenolic content assays, Folin–Ciocalteu solution, dimethylsulfoxide (DMSO), sodium carbonate (Na_2_CO_3_), gallic acid, and 2,2-diphenyl-1-picrylhydrazyl (DPPH) were purchased from Sigma-Aldrich (Sigma-Aldrich, Steinheim, Germany). The n-alkanes C9-C30 for Kovats index (KI) were supplied from Fluka (Fluka Chemie, Buchs, Switzerland).

### 2.2 Plant material and extra virgin olive oil

*Origanum vulgare* subsp. *hirtum* L. (oregano) and *Thymus vulgaris* L. var. Varico 3 (thyme), as well as by–products of their processing, were studied. Plant propagating material was originated by mother plants maintained *ex-situ*, at the collection of Balkan Botanic Garden of Kroussia (41°05′44.3”N 23°06′33.7″E) of the Institute of Plant Breeding and Genetic Resources, Hellenic Agricultural Organization (ELGO)—DIMITRA, in Greece. The aerial parts of each plant were collected, dried, and processed in a grinding mill to separate leaves and flowers from branches. Simultaneously, the mill automatically separated the grated plant material into grades determined according to Codex Alimentarius (CX/SCH 15/02/06 for oregano and CX/SCH 15/02/07 for thyme). After drying and processing, the quantities of first (A, 4.5–1.0 mm) and second (B, 1.0–0.5 mm) grade, as well as the post harvesting by-products (W, aerial parts remaining biomass) were measured. Qualities A and B are commercially acceptable from the market (superior plant material) while the W quality is considered non-commercial (inferior plant material). Plant material propagation and plant sampling details can be found in previous research (12). Extra virgin olive oil was produced from Koroneiki variety from olive trees cultivated in Crete, Greece.

### 2.3 Extraction procedures

**Figure 1:**
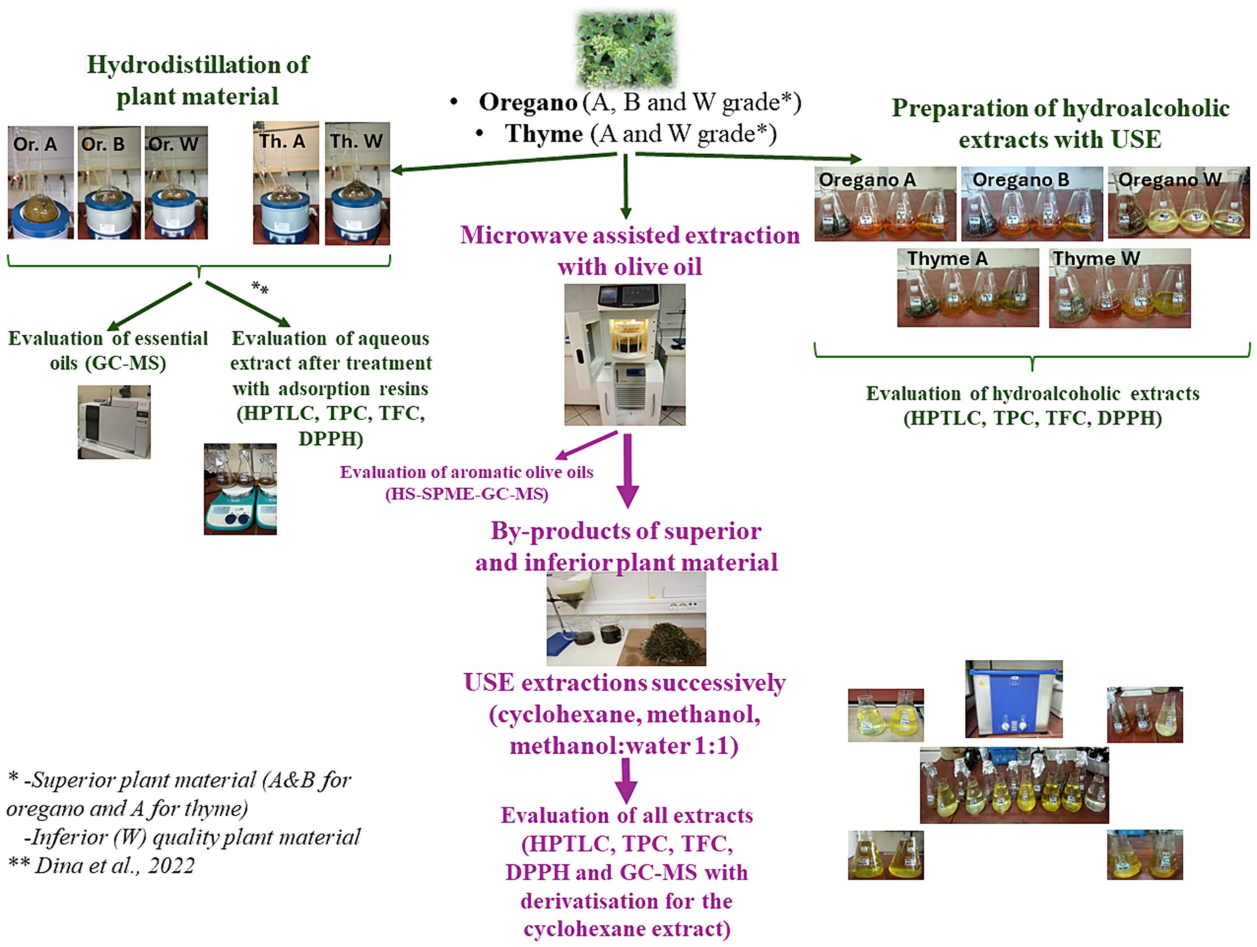
Scheme of extraction procedures, chemical analysis and evaluation of the superior (A&B for oregano and A for thyme) and inferior (W) plant materials.

All extraction procedures followed for the evaluation of the superior (A&B for oregano and A for thyme) and inferior (W) plant materials can be found in Figure 1.

#### 2.3.1 Hydrodistillation (HD) and essential oils (EO) productions

The collected plant material (thyme and oregano) was subjected to hydrodistillation, to afford the EOs. For this reason, 100 g of aerial parts from superior (A&B for oregano and A for thyme) and inferior (W) quality of the plants were distilled using 1,000 mL water at 100°C for 3 h in a Clevenger apparatus. The percentage yield (v/w) of the produced EOs was estimated, while they were dried over sodium sulfate anhydrous and stored at 4°C until they were analyzed.

#### 2.3.2 Microwave assisted extraction (MAE)

Superior (A&B for oregano and A for thyme) and inferior (W) quality plant material were extracted with extra virgin olive oil using a Milestone NEOS-GR microwave unit (Milestone Srl, Sorisole, Italy). 50 g of each pulverized plant material were extracted with 1,500 mL of extra virgin olive oil. The plant material with the olive oil were heated gradually for 10 min at 60°C and then remained for 15 min at 60°C. The upper limit of the extraction temperature was 64°C. From this procedure, apart from the olive oil extracts, the remaining plant material was stored (−10°C) for further analysis.

#### 2.3.3 Ultrasound assisted extraction (UAE)

Hydroalcoholic extracts of superior (A and B grade) and inferior quality (W grade) plant material (thyme, oregano) were produced using the Elma S 100 H ultrasound assisted extraction (UAE) apparatus (Elmasonic, Singen, Germany). 5 g of each pulverized plant material were extracted with 200 mL of a hydro-alcoholic mixture (H_2_O:MeOH 50:50) in 3 consecutive cycles for 30 min at 35–40°C. Solvents were evaporated under reduced pressure using a rotary evaporator (Büchi Labortechnik AG, Flawil, Switzerland) and percentage yield (w/w) for every extract was estimated. In total, 3 extracts from oregano (A, B and W grade) and 2 extracts from thyme (A and W grade) were produced, stored (−10°C) and subjected to further analysis.

Moreover, the remaining plant material obtained after the olive oil aromatization by MAE was further processed. 10 g of each plant material was extracted with 200 mL of c-Hex, MeOH and H_2_O:MeOH 50:50 successively with UAE apparatus. All the plant materials were extracted in 2 consecutive cycles for each solvent, for 30 min at 35–40°C. Solvents were evaporated under reduced pressure. In total, 9 extracts (3 c-Hex, 3 MeOH and 3 H_2_O:MeOH 50:50) were produced, stored (−10°C) and subjected to further analysis.

### 2.4 Gas chromatography–mass spectrometry (GC–MS) analysis

The identification of the chemical composition of the EOs was performed with an Agilent 7820A Gas Chromatograph System linked to Agilent 5977B mass spectrometer system (Agilent Technologies, Santa Clara, CA, United States) equipped with a HP5-MS capillary column (30 m × 0.25 mm and 0.25 μm film thickness). The initial column temperature was 60°C and then increased at a rate of 3°C/min to a maximum temperature of 300°C, where it remained for 10 min. Total analysis time was 90 min. Helium was used as a carrier gas at a flow rate of 1.0 mL/min, split ratio 1:10, injector temperature 220°C, and ionization voltage 70 eV. The compound identification was conducted using the NIST14 and ADAMS 07 libraries, bibliographic data, and the comparison of the Kovats (IK) and Adams indices. The Kovats indices compare the retention time of a product with a linear alkane of the same number of carbons and were determined by injecting a mixture of alkanes (standard C9–C30) under the same operating conditions. The chromatograms were processed with Agilent MSD Chemstation Data Analysis software.

Furthermore, the analysis of the c-Hex extracts of the remaining plant material obtained after the olive oil aromatization by MAE, was performed with the same GC–MS apparatus using the following method: the initial column temperature was 60°C, remaining for 5 min, and then increased at a rate of 5°C/min to a maximum temperature of 260°C, where it remained for 5 min. Finally, the temperature was increased to 280°C in 10 min. Total analysis time was 57 min. Prior to analysis, in order to transform the fatty acids into their respective volatile methyl esters, a derivatization procedure was carried out. Briefly, a solution of 5 mg/mL of c-Hex extracts was prepared and 200 μL of potassium hydroxide in methanol (2 M) was added, followed by shaking. The supernatants of the biphasic system occurred for every sample were injected to GC–MS for further analysis.

### 2.5 Headspase solid phase microextraction—(HS-SPME) procedure

The extra virgin olive oil and the obtained aromatic oils were analyzed with HS-SPME-GC–MS. The SPME fiber (divinylbenzene-DVB/carboxen-CAR/polydimethylsiloxane-PDMS, 50/30 um) and SPME fiber holder for manual sampling were purchased from Supelco (Sigma-Aldrich, Bellefonte, PA, USA). Before analysis, the fiber was conditioned for 15 min at 250°C. For each extraction, 2 g of sample was placed into a 10 mL amber vial closed with a PTFE/silicone septum (Supelco) and 0.2 g of NaCl for 60 min at 40°C under constant stirring (750 rpm). After extraction, the fiber was introduced into the GC system injection port at 250°C for 6 min (Section 2.4), where the analytes were thermally desorbed from the fiber coating and transferred directly to the GC system column. The sample was injected in a spitless mode. The initial column temperature was 40°C, then increased at a rate of 4°C/min until 75°C, and finally at a rate of 8°C/min to a maximum temperature of 250°C, where it remained for 5 min. Total analysis time was 35.6 min. The compound identification was conducted using the NIST14 and ADAMS 07 libraries, bibliographic data and the comparison of the kovats (IK) and Adams indices.

### 2.6 High performance thin layer chromatography (HPTLC) analysis

The chemical profile of the extracts obtained from all aforementioned procedures was determined using High Performance Thin Layer Chromatography (HPTLC), purchased from Camag (CAMAG, Muttenz, Switzerland). Samples were applied on silica gel F254-precoated plates from Merck (Merck, Darmstadt, Germany) using an automated sample applicator ATS4 and the chromatograms were developed in an ADC2 automated development chamber with the appropriate mobile phase. The plates were documented under UV 254 and 366 nm and after spraying with sulfuric vanillin using the TLC Visualizer 2. The system was operating under the VisionCats 3.0 software. For the HPTLC fingerprinting, 100 μg of each sample were loaded on normal and reversed phase TLC plates and solvent mixtures of EtOAc:MeOH:A.A (70:30:1) and H_2_O:ACN:A.A (80:20:1) were used, respectively, as a mobile phase.

### 2.7 Total phenolic content (TPC) and 2,2-diphenyl-1-picrylhydrazyl (DPPH) free radical scavenging assays

The evaluation of the Total Phenolic Content (TPC) and 2,2-diphenyl-1-picrylhydrazyl (DPPH) free radical scavenging activity of the hydroalcoholic (5 samples: A, B and W for thyme, and A&W for oregano) and after the extraction with olive oil (15 samples: c-Hex, MeOH and H2O:MeOH for oregano and thyme superior & inferior) extracts was performed as described in Dina et al. (12). Shortly, Folin–Ciocalteu solution for TPC assay was prepared with 10% dilution in distilled water and the alkaline environment was achieved with the addition of 7.5% sodium carbonate in distilled water. Extracts were prepared using dimethylsulfoxide (DMSO) as a solvent in stock concentrations and dilutions were made if necessary. In 96 well plates, 25 μL of extract in dimethyl sulphoxide (DMSO), 125 μL Folin–Ciocalteu solution and 100 μL Na_2_CO_3_ solution were mixed. The plates were incubated for 30 min at ambient temperature in the dark. Absorbance was measured at 765 nm, using a microplate reader (Tecan, Männedorf, Switzerland). TPC was expressed as mg of gallic acid equivalent per g of dried extract (mg GAE/g dry weight). Each sample was tested in triplicate.

Evaluation of the free radical scavenging activity of all extracts was performed using the free radical 2,2-diphenyl-1-picrylhydrazyl (DPPH) assay. Extracts were prepared using dimethylsulfoxide (DMSO) as a solvent in an initial concentration of 4 mg/mL (stock solution) and dilutions were made to reach the tested concentrations (200 and 100 μg/mL in the well). 10 μL of each extract in DMSO and 190 μL of DPPH solution (12.4 mg/100 mL in ethanol) were mixed in a 96-well plate and then subsequently incubated, at room temperature, for 30 min in darkness. Finally, the absorbance was measured at 517 nm in a microplate reader (Tecan, Männedorf, Switzerland). All evaluations were performed in triplicates, gallic acid was used as positive control and the % inhibition of the DPPH radical was estimated by the following equation:


[(A−B)−(C−D)]/(A−B)×100,

where A: Control (w/o sample), B: Blank (w/o sample, w/o DPPH), C: sample, D: Blank sample (w/o DPPH).

## 3 Results

### 3.1 Analysis of volatile compounds

#### 3.1.1 Hydrodistillation and GC–MS analysis of EOs

In our previous research, for the evaluation of raw plant material, hydrodistillation process was employed to receive the EOs of superior and inferior plant material. In this study, commercially acceptable plant material of grade B (1.0–0.5 mm) oregano was further evaluated. After hydrodistillation, oregano grade B quality resulted in similar EOs %yield (ORV2_HDEO 3.4% v/w) with grade A (ORV1_HDEO 4% v/w), while as previously mentioned, EOs of grade W for oregano and thyme resulted in lower yields (Supplementary Table S1).

GC–MS analyses of oregano and thyme EOs from superior and inferior plant material are shown in Supplementary Table S2 and Supplementary Figures S1–S5. In oregano EO obtained from grade B raw material, 19 constituents were identified representing 99.82% of the total content. The major compounds were carvacrol (72.42%), *p*-cymene (7.62%), thymol (7.15%), and *γ*-terpinene (2.69%). In the case of carvacrol and *p*-cymene, their percentage were slightly higher (72.42 and 7.62%, respectively) compared to oil obtained from grade A raw material (64.78 and 4.29%, respectively). Other compounds present in oregano grade B were: *trans-*caryophyllene, *β-*bisabolene, caryophyllene oxide, *δ*-2-carene, carvacrol methyl ether, *a*-pinene, *β*-myrcene, borneol, terpinen-4-ol, 1-octen-3-ol, *β*-phellandrene, *trans*-sabinene hydrate.

#### 3.1.2 Microwave assisted extraction (MAE) with extra virgin olive oil and HS-SPME-GC–MS analysis of aromatized olive oils

Oregano and thyme superior and inferior plant material were extracted with extra virgin olive oil by microwave assisted extraction (MAE) to afford aromatized olive oil extracts. The extra virgin olive oil and the obtained aromatic olive oils were analyzed with HS-SPME-GC–MS and the respective chromatograms can be found in Supplementary Figures S6–S11. After analyses, 8 constituents were identified in the aromatic olive oil obtained with oregano superior plant material (ORV1_AOO and ORV2_AOO) representing 81.93 and 84.96% of the total content (Table 1) for grade A and B, respectively, whereas no constituents were identified in the aromatized olive oil obtained with inferior raw material. The major constituents of oregano olive oil were carvacrol, *p*-cymene and *γ*-terpinene and *β*-myrcene, which were detected in both grade A and grade B in similar percentages (Table 1).

**Table 1:** Chemical composition of aromatized olive oils deriving from oregano and thyme superior plant material after HS-SPME-GC–MS analysis.

		ORV1_AOO	ORV2_AOO	THV_AOO
KI	Constituents	Area %	Area %	Area %
930	*α*-thujene	2.92	2.56	2.39
952	*α*-pinene	1.00	1.04	–
981	1-octen-3-ol	2.27	2.32	2.40
992	*β*-myrcene	8.15	8.01	5.64
1022	*p*-cymene	18.82	24.21	22.72
1055	*γ*-terpinene	15.09	10.92	24.16
1070	*trans*-sabinene hydrate	–	–	1.65
1160	borneol	1.07	1.12	5.88
1291	thymol	–	–	17.27
1306	carvacrol	32.61	34.78	1.46
Total %		81.93	84.96	83.57

ORV1_AOO, aromatized olive oil of oregano grade A; ORV2_AOO, aromatized olive oil of oregano grade B; THV_AOO, aromatized olive oil of thyme grade A.

Correspondingly, in the case of the analyses of thyme olive oil, 9 constituents were identified in olive oil obtained from superior plant material (THV_AOO) representing 83.57% of the total content (Table 1). The major constituents detected were thymol, *p-*cymene, carvacrol, *γ-*terpinene *β-*myrcene and borneol. As in the case of oregano, no constituents were identified in the thyme inferior quality. It is notable that, HS-SPME-GC–MS analyses revealed the presence of their corresponding EOs major compounds (carvacrol, thymol, *γ-*terpinene and *p-*cymene), underlying the high added-value of the enriched aromatic olive oils.

### 3.2 Ultrasound assisted extraction (UAE)

For the evaluation of raw plant material, beside the characterization of the EOs, the hydroalcoholic extracts of superior and inferior plant material were prepared by UAE and their percentage yield was calculated (Supplementary Table S3). In the case of grade B (1.0–0.5 mm) oregano the yield was lower (19.3% w/w) in comparison to grade A (30.6%), while the inferior plant material of grade W resulted in an extract with the smaller yield (4.10%).

In addition, the plant material was used to afford aromatized olive oils after MAE. The remaining plant by-products were extracted by UΑE to be further analyzed. In Table 2, % yield (w/w) of by-product extracts after UΑE are shown.

**Table 2:** % yield (w/w) of MAE by-product extracts and after ultrasound assisted extraction.

Plant species	Plant material	Code	%yield (w/w)
*UAE extracts of remaining plant material after olive oil MAE*			
Oregano (c-Hex)	Superior (grade A)	ORV1_MAE_HEX	43.4
	Superior (grade B)	ORV2_MAE_HEX	49.2
	Inferior (grade W)	ORVW_MAE_HEX	9.8
Thyme (c-Hex)	Superior (grade A)	THV_MAE_HEX	45.4
	Inferior (grade W)	THVW_MAE_HEX	73.5
Oregano (MeOH)	Superior (grade A)	ORV1_MAE_MEOH	6.8
	Superior (grade B)	ORV2_MAE_MEOH	4.0
	Inferior (grade W)	ORVW_MAE_MEOH	1.9
Thyme (MeOH)	Superior (grade A)	THV_MAE_MEOH	3.6
	Inferior (grade W)	THVW_MAE_MEOH	5.9
Oregano (H_2_O:MeOH 50:50)	Superior (grade A)	ORV1_MAE_WM	12.1
	Superior (grade B)	ORV2_MAE_WM	10.2
	Inferior (grade W)	ORVW_MAE_WM	1.7
Thyme (H_2_O:MeOH 50:50)	Superior (grade A)	THV_MAE_WM	5.2
	Inferior (grade W)	THVW_MAE_WM	3.3

### 3.3 High performance thin layer chromatography (HPTLC) profiling of extracts

**Figure 2:**
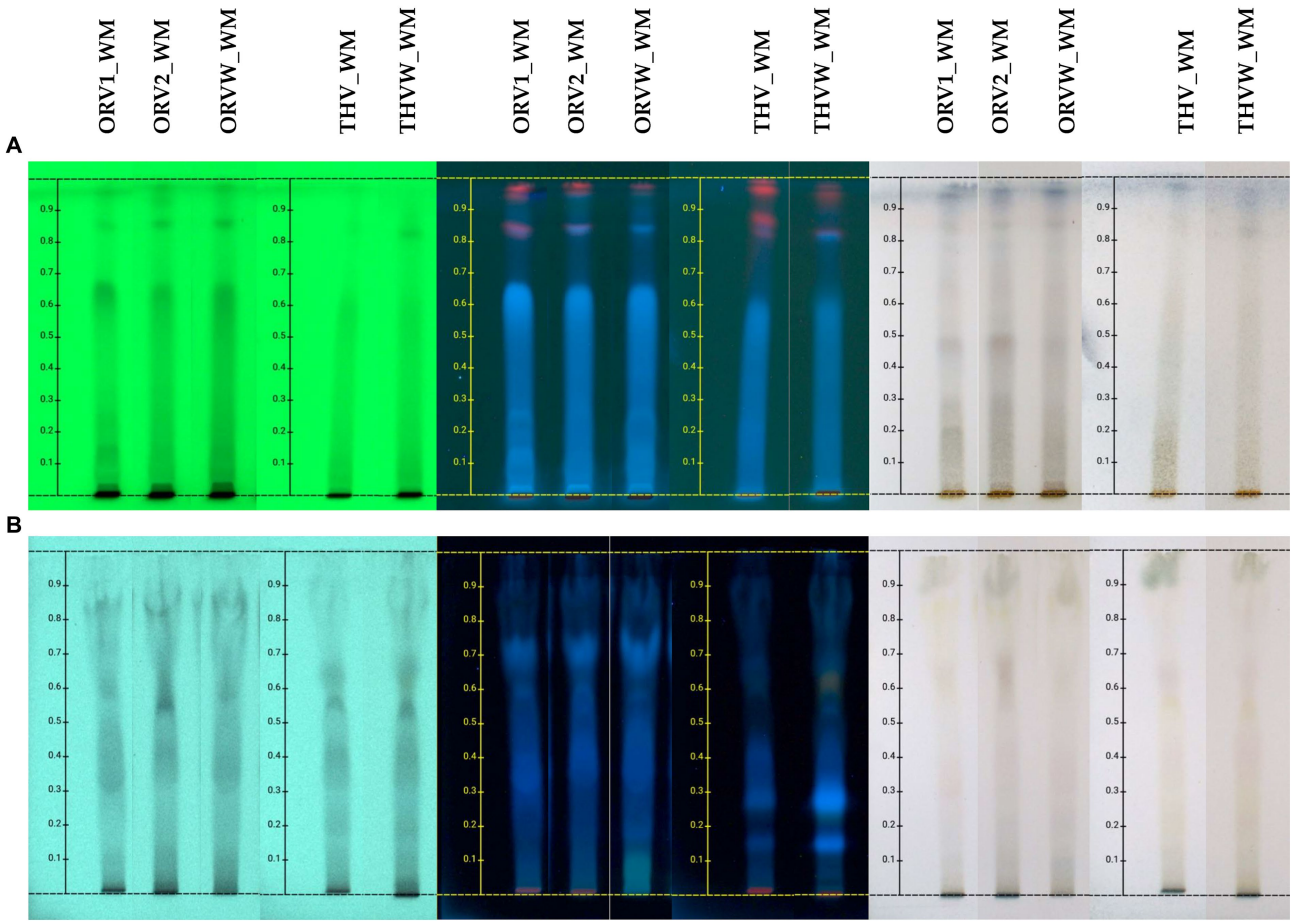
HPTLC chromatograms of hydroalcoholic extracts of oregano [superior plant material: ORV1 (grade A), ORV2 (grade B); inferior plant material: ORVW] and thyme (superior plant material: THV; inferior plant material: THVW), on normal **(A)** and reversed **(B)** phase TLC plates.

The chemical profile of hydroalcoholic extracts of raw plant material and extracts of remaining plant material after olive oil flavoring was investigated using the HPTLC technique. HPTLC results (Figure 2) revealed the presence of similar secondary metabolites in the extracts obtained from both superior and inferior raw materials for all the studied herbs. Oregano grade B (ORV2_WM) was mostly characterized by the presence of terpenoids (no absorbance under UV light at 254 nm and blue-purple spots after spraying and heating), phenolics (absorbance at 254 nm and orange/pink spots after spraying with vanillin-sulfuric acid solution and heating), and sugars (no absorbance, dark grey spots after spraying and heating) (13), as oregano grade A.

**Figure 3:**
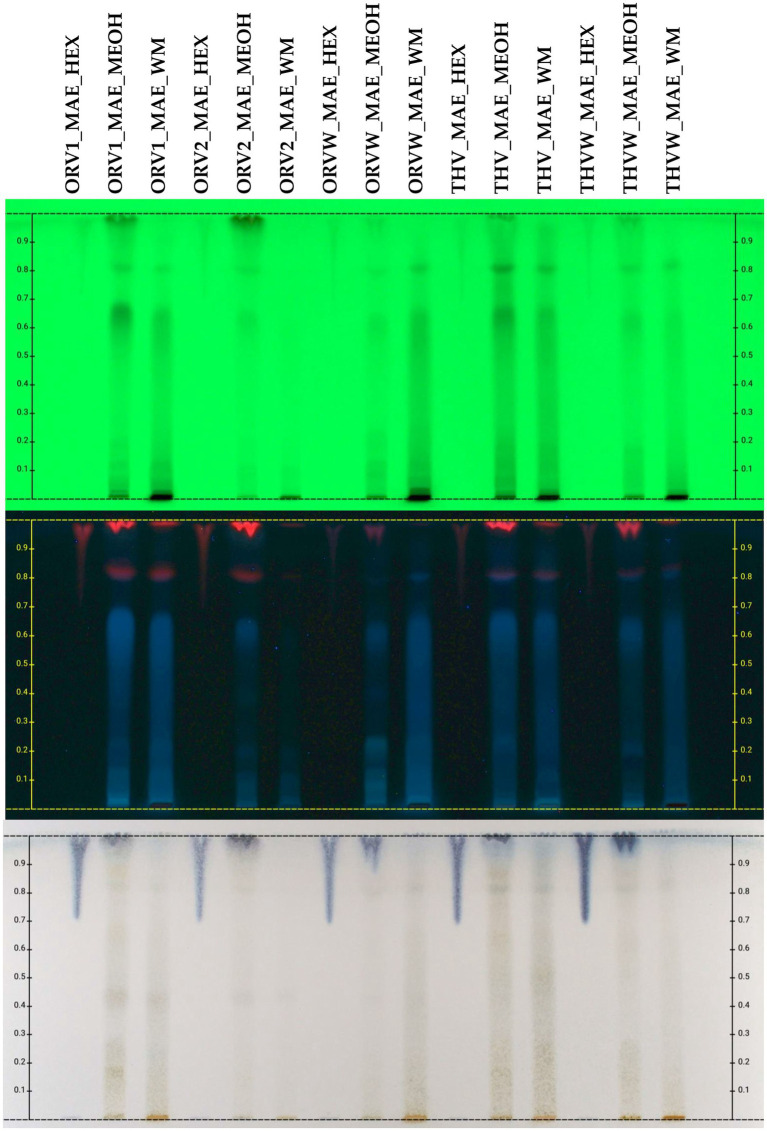
HPTLC chromatograms of UAE extracts (HEX: cyclohexane, MEOH: methanol, WM: H_2_O:MeOH 50:50) of plant remaining by-products after MAE of oregano [superior plant material: ORV1 (grade A), ORV2 (grade B); inferior plant material: ORVW] and thyme (superior plant material: THV; inferior plant material: THVW), on normal phase TLC plate.

**Figure 4:**
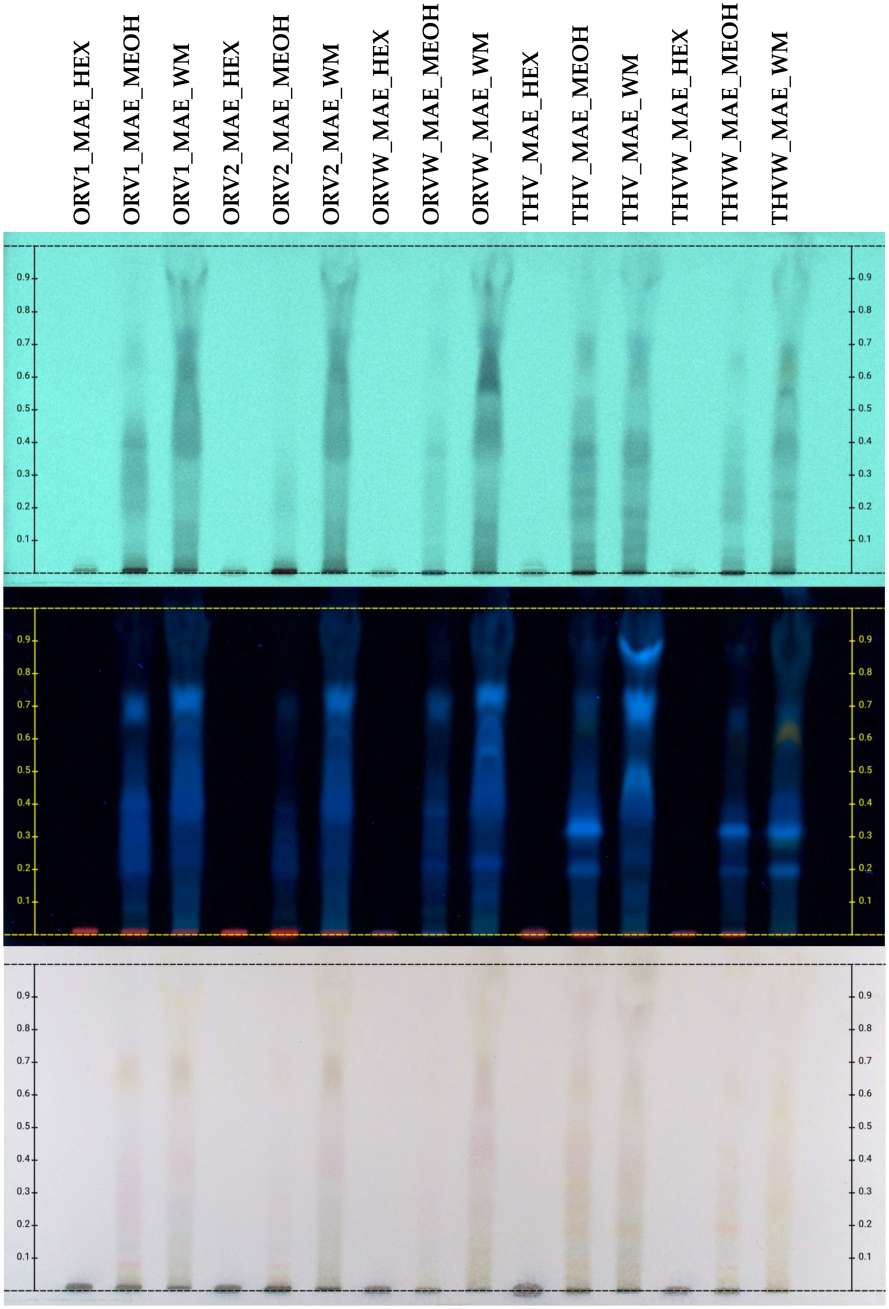
HPTLC chromatograms of UAE extracts (HEX: cyclohexane, MEOH: methanol, WM: H_2_O:MeOH 50:50) of plant remaining by-products after MAE of oregano [superior plant material: ORV1 (grade A), ORV2 (grade B); inferior plant material: ORVW] and thyme (superior plant material: THV; inferior plant material: THVW), on reversed phase TLC plate.

The chemical profile of cyclohexane (c-Hex), methanolic (MeOH) and hydroalcoholic (H_2_O:MeOH 50:50) extracts was also investigated by HPTLC in normal (Figure 3) and reversed (Figure 4) phase plates. The chemical profile of c-Hex extracts was analyzed by HPTLC, but no satisfactory fingerprints were obtained due to the presence of oil as expected. However, GC–MS analysis of c-Hex extracts (Supplementary Figures S12–S17) revealed only the presence of olive oil constituents (Supplementary Table S5) such as 9-hexadecenoic acid methyl ester (0.77%), hexadecanoic acid methyl ester (12.88%), 9,12-octadacenoic acid methyl ester (6.57%), 9-octadacenoic acid methyl ester (63.72%), methyl stearate (2.31%) indicating that flavoring with MAE under the described conditions is not destructive for the olive oil. The HPTLC fingerprint of methanolic extracts demonstrated quantitative differences for superior (grade A and B) and inferior plant materials (grade W) for oregano and thyme, while the profile of the hydroalcoholic extracts was similar for superior and inferior qualities. Moreover, the sugar content of the hydroalcoholic extracts, as shown in reversed phase HPTLC, was remarkably higher than the methanolic ones. Finally, HPTLC results revealed that the methanolic (MeOH) and hydroalcoholic (H_2_O:MeOH 50:50) extracts were mostly characterized by the presence of terpenoids (no absorbance under UV light at 254 nm and blue-purple spots after spraying and heating), flavonoids (absorbance at 254 nm and orange/yellow spots after spraying with vanillin-sulfuric acid solution and heating), and sugars (no absorbance, dark grey spots after spraying and heating, increased polarity) (13).

### 3.4 Total phenolic content (TPC) and DPPH and 2,2-diphenyl-1-picrylhydrazyl (DPPH) free radical scavenging activity of extracts

The total phenolic content and DPPH free radical scavenging activity of all extracts was measured and the results of hydroalcoholic extracts of superior and inferior plant material are shown in Supplementary Table S4. In the case of oregano, both categories of superior raw materials were characterized by similar levels of phenolic compounds and DPPH inhibition. Extracts obtained from the oregano grade B showed slightly lower levels of phenols (151.3 mg GAE/g dw) and DPPH inhibition (79.2, 78.1 and 53.8% at 200, 100 and 50 μg/mL final concentration, respectively) in comparison to grade A (160.1 mg GAE/g dw and DPPH: 91.5, 87.1 and 60.3% at 200, 100 and 50 μg/mL final concentration, respectively) (12).

In addition, the methanolic and hydroalcoholic extracts of the plant by-products after MAE extraction with olive oil were evaluated (Table 3). For oregano, results revealed no differences between MeOH and H_2_O:MeOH extracts obtained from superior and inferior quality plant material in regard to TPC and DPPH inhibition. Interestingly, hydroalcoholic MAE by-products extracts showed similar phenolic content (ORV1_WM: 160.1 vs. ORV1_MAE_WM: 170.5 mg GAE/g dw; ORV2_WM: 151.3 vs. ORV2_MAE_WM: 151.95 mg GAE/g dw; ORVW_WM: 143.8 mg vs. ORVW_MAE_WM: 143.8 mg GAE/g dw) and DPPH scavenging capacity (ORV1_WM: 87.1% vs. ORV1_MAE_WM: 82.8%; ORV2_WM: 78.1% vs. ORV2_MAE_WM: 84.5%; ORVW_WM: 78.1% vs. ORVW_MAE_WM: 79.8% at 100 μg/mL) with hydroalcoholic extracts from raw plant material, as shown in Supplementary Table S4 and Table 3.

**Table 3:** Total phenolic content and DPPH free scavenging capacity of UAE extracts of remaining plant material after olive oil MAE.

Plant species	Plant material	Code	% DPPH inhibition			TPC
			200 μg/mL	100 μg/mL	50 μg/mL	mg GAE/g dry weight
*UAE extracts of remaining plant material after olive oil MAE*						
Oregano (c-Hex)	Superior (grade A)	ORV1_MAE_HEX	ND	ND	ND	ND
	Superior (grade B)	ORV2_MAE_HEX	ND	ND	ND	ND
	Inferior (grade W)	ORVW_MAE_HEX	ND	ND	ND	ND
Thyme (c-Hex)	Superior (grade A)	THV_MAE_HEX	ND	ND	ND	1.18 ± 1.8
	Inferior (grade W)	THVW_MAE_HEX	ND	ND	ND	ND
Oregano (MeOH)	Superior (grade A)	ORV1_MAE_MEOH	69.91 ± 4.9	33.14 ± 0.6	12.37 ± 0.4	53.42 ± 3.5
	Superior (grade B)	ORV2_MAE_MEOH	54.13 ± 4.67	24.52 ± 0.7	8.52 ± 0.7	38.45 ± 2.2
	Inferior (grade W)	ORVW_MAE_MEOH	35.66 ± 0.7	17.89 ± 0.8	5.89 ± 0.5	26.29 ± 1.4
Thyme (MeOH)	Superior (grade A)	THV_MAE_MEOH	90.49 ± 0.2	51.44 ± 4.2	42.39 ± 11.7	98.35 ± 4.9
	Inferior (grade W)	THVW_MAE_MEOH	24.46 ± 1.0	10.12 ± 0.2	ND	16.53 ± 0.9
Oregano (H_2_O:MeOH 50:50)	Superior (grade A)	ORV1_MAE_WM	82.35 ± 0.4	82.83 ± 0.4	49.78 ± 0.5	170.53 ± 12.49
	Superior (grade B)	ORV2_MAE_WM	81.59 ± 0.4	84.51 ± 1.5	48.60 ± 0.5	151.95 ± 9.5
	Inferior (grade W)	ORVW_MAE_WM	81.99 ± 0.4	79.88 ± 0.4	52.54 ± 0.1	143.81 ± 4.8
Thyme (H_2_O:MeOH 50:50)	Superior (grade A)	THV_MAE_WM	90.49 ± 0.2	75.03 ± 1.0	22.26 ± 0.5	115.73 ± 2.2
	Inferior (grade W)	THVW_MAE_WM	83.0 ± 0.3	78.03 ± 1.2	40.30 ± 2.1	127.06 ± 12.8

In the case of thyme, hydroalcoholic MAE by-products extracts showed in-between them similar phenolic content and slightly lower in comparison to the UAE ones (THV_WM: 177.2 vs. THV_MAE_WM: 115.7 mg GAE/g dw; THVW_WM: 166.4 vs. THVW_MAE_WM 127.0 mg GAE/g dw). All extracts exhibited similar DPPH scavenging capacity (THV_WM: 85.9% vs. THV_MAE_WM: 90.5%; THVW_WM: 82.7% vs. THVW_MAE_WM: 83.0% at 200 μg/mL) with hydroalcoholic extracts from raw plant material.

## 4 Discussion

Oregano (*Origanum vulgare* subsp. *hirtum* L.) and thyme (*Thymus vulgaris* L.) are among the most popular herbs in Mediterranean countries, and in Greece are traditionally used for their medicinal properties but also for culinary purposes. Moreover, these plant species are utilized for the flavoring of extra virgin olive oil to produce enriched aromatic olive oils. In this study, different grades (superior and inferior) of plant material were evaluated for their potential use in the aromatization of olive oil by microwave assisted extraction (MAE). It was clearly demonstrated that post-harvesting non-commercially accepted (inferior) plant material of oregano and thyme cannot be utilized for the direct aromatization of olive oil by MAE, even though hydrodistillation of the same material can afford a limited quantity of EOs (Table 1; Supplementary Table S2). However, in the case of oregano, aerial parts counted as B grade (1.0–0.5 mm) could be used for the aromatization of olive oils, since the expected volatile compounds were present in the final product as shown by HS-SPME-GC–MS analysis (Table 1).

Continuously, the second aim of this study was to evaluate the MAE extractions by-products, meaning the plant material remaining after the extraction of the superior and inferior quality of oregano and thyme with olive oil. In particular, plant material was extracted successively with c-Hex, MeOH and (H_2_O:MeOH) 50:50. The c-Hex extracts, after GC–MS and HPTLC analysis, showed poor chemical profile, consisting mainly of the olive oil fatty constituents (Supplementary Table S5; Supplementary Figures S12–S17). However, this first extraction step with c-Hex proved to be a prerequisite for further processing of the plant material and it can be considered as a crucial defatting step. Then, the methanolic and hydroalcoholic extracts were evaluated for their total phenolic content (TPC) and DPPH free scavenging activity and were chemically screened by means of HPTLC. According to the results, methanolic extracts obtained from grade A and B of oregano revealed similar HPTLC fingerprints, while the DPPH inhibition and TPC content was slightly lower in grade B (69.91 and 54.13% DPPH inhibition at 200 μg/mL; 53.42 and 38.45 mg GAE/gr dw, for A and B, respectively) in comparison to those of inferior plant material that was lower (35.66% DPPH inhibition at 200 μg/mL and 26.29 mg GAE/gr dw, respectively). That was not the case for thyme, where inferior plant material did not show any biological activity. Finally, the hydroalcoholic extracts of both plant species showed similar HPTLC fingerprints, DPPH inhibition and TPC with extracts obtained from the inferior plant material, something that was demonstrated also for the raw plant material (Table 3).

To conclude, in our previous research (12) it was demonstrated that post-harvesting by-products revealed similar chemical profile as well as strong antioxidant capacity which was related to the detected high phenolic content, indicating their usefulness as food additives. This study revealed the presence of EOs’ major compounds (carvacrol, thymol, *γ-*terpinene, *p-*cymene, etc.) of the respective raw plant material. The use of these plant materials showed similar content with grade A quality during the aromatization and it was clearly demonstrated that they can be used for the production of enriched aromatic olive oils with high added value. Finally, the plant material remaining after aromatization can be further exploited since the hydroalcoholic extracts obtained from MAE extraction by-products showed similar phenolic content and scavenging activity with the hydroalcoholic extracts from the raw plant material. These results highlight that the aforementioned extracts are rich in bioactive constituents and can be utilized to develop novel products such as cosmeceuticals and/or nutraceuticals.

## Data availability statement

The original contributions presented in the study are included in the article/Supplementary material, further inquiries can be directed to the corresponding author.

## Author contributions

ED: Data curation, Formal analysis, Investigation, Methodology, Software, Visualization, Writing – original draft. AC: Data curation, Formal analysis, Investigation, Methodology, Software, Supervision, Visualization, Writing – original draft. AV: Formal analysis, Investigation, Methodology, Software, Writing – review & editing. DK: Investigation, Writing – review & editing. ID: Funding acquisition, Resources, Supervision, Writing – review & editing. PB: Funding acquisition, Resources, Supervision, Writing – review & editing. IG: Resources, Supervision, Writing – review & editing. KG: Conceptualization, Funding acquisition, Methodology, Resources, Supervision, Writing – review & editing. NA: Conceptualization, Funding acquisition, Methodology, Resources, Supervision, Writing – review & editing.
