# Supplementary material for: Oregano and thyme by-products of olive oil aromatization process with microwave assisted extraction as a rich source of bio-active constituents
Source: Front Nutr. 2024 May 9;11:1372263. doi: 10.3389/fnut.2024.1372263 (PMC11111879; doi:10.3389/fnut.2024.1372263)
Supplement: Supplementary file 1 [file Table_1.DOCX]

Supplementary Material

Oregano and thyme by-products of olive oil aromatization process with microwave assisted extraction as a rich source of bio-active constituents.

Evanthia Dina^1^, Antigoni Cheilari^1,*^, Argyro Vontzalidou^1^, Karamani Dimitra^2^, Diamanti Ioanna^2^, Panagiotis Bagatzounis^3^, Ilias Giannenas^4^, Katerina Grigoriadou^5^, Nektarios Aligiannis^1^

^1^Department of Pharmacognosy and Natural Products Chemistry, Faculty of Pharmacy, National and Kapodistrian University of Athens, Panepistimiopolis Zografou, Athens 15771, Greece

^2^Pellas Nature S.A, Seremes, Proastio, 58200. Edessa Greece

^3^Bagatzounis & Sons S.A, Vatero, 50100, Kozani, Greece

^4^Laboratory of Nutrition, School of Veterinary Medicine, AUTH, 54124, Thessaloniki, Greece

^5^Institute of Plant Breeding and Genetic Resources, Hellenic Agricultural Organization – DEMETER, P.O. Box 60458, 570 01 Thermi, Thessaloniki, Greece

***Correspondence:** Antigoni Cheilari, cheilarianti@pharm.uoa.gr

Keywords: aromatization, aromatized olive oil, oregano, thyme, microwave assisted extraction, ultrasound assisted extraction, plant by-products, enriched extracts

# Supplementary Data

**Table S1.** % yield (v/w) of essential oils deriving from oregano and thyme superior and inferior plant material.

| **Plant species** | **Plant material** | **Code** | **% EO yield (v/w)** |
| --- | --- | --- | --- |
| Oregano  (*Origanum vulgare* subsp. *hirtum* L.) | Superior (grade A) | **ORV1_HDEO** | 4.0% |
|  | Superior (grade B) | **ORV2_HDEO** | 3.4% |
|  | Inferior (grade W) | **ORVW_HDEO** | 0.8% |
| Thyme  (*Thymus vulgaris* L.) | Superior (grade A) | **THV_HDEO** | 1.2% |
|  | Inferior (grade W) | **THVW_HDEO** | 0.15% |

**Table S2** Chemical composition of essential oils of superior and inferior plant material of oregano (superior plant material: ORV1_HDEO, ORV2_HDEO; inferior plant material: ORVW_HDEO:) and thyme (superior plant material: THV_HDEO; inferior plant material: THVW_HDEO:).

|  |  | **ORV1_HDEO** | **ORV2_HDEO** | **ORVW_HDEO** | **THV_HDEO** | **THVW_HDEO** |
| --- | --- | --- | --- | --- | --- | --- |
| **KI** | **Constituents** | **Area %** | | | | |
| 952 | ***α*-pinene** | 0.40 | 0.44 | - | 0.90 | - |
| 962 | **camphene** | 0.09 | 0.12 | - | 0.77 | - |
| 981 | **1-octen-3-ol** | 0.25 | 0.27 | - | 0.60 | - |
| 992 | ***β*-myrcene** | 0.57 | 0.39 | 0.65 | 0.34 | 0.44 |
| 1003 | ***α*-phellandrene** | 0.09 | - | 0.10 | - | - |
| 1014 | ***δ*-2-carene** | 0.71 | 0.56 | 0.72 | 0.79 | 0.68 |
| 1022 | ***p*-cymene** | 4.29 | 7.62 | 6.68 | 22.50 | 21.88 |
| 1025 | **limonene** | - | - | - | 0.33 | 0.21 |
| 1025 | ***β*-phellandrene** | 0.23 | 0.26 | 0.28 | - | - |
| 1027 | **1,8-cineol** | - | - | - | 0.61 | 0.43 |
| 1055 | ***γ*-terpinene** | 4.00 | 2.69 | 2.13 | 4.43 | 2.00 |
| 1070 | ***trans*-sabinene hydrate** | 0.22 | 0.20 | 0.17 | 0.15 | 0.16 |
| 1088 | **terpinolene** | - | - | - | 0.15 | 0.12 |
| 1099 | **linalool** | 0.13 | 0.12 | - | 1.30 | 1.62 |
| 1138 | **camphor** | - | - | - | 0.51 | 1.73 |
| 1160 | **borneol** | 0.39 | 0.34 | 0.30 | 1.71 | 1.69 |
| 1173 | **terpinen-4-ol** | 0.47 | 0.32 | 0.61 | 0.66 | 0.72 |
| 1186 | ***α*-terpineol** | - | - | - | 0.22 | 0.23 |
| 1235 | **thymol methyl ether** | - | - | - | 1.23 | 1.36 |
| 1239 | **carvacrol methyl ether** | 0.27 | 0.47 | - | 0.84 | 0.40 |
| 1291 | **thymol** | 20.14 | 7.15 | 7.19 | 53.04 | 56.50 |
| 1306 | **carvacrol** | 64.78 | 72.42 | 78.20 | 5.15 | 7.25 |
| 1351 | **eugenol** | - | - | 0.21 | 0.12 | 0.09 |
| 1412 | ***trans*-caryophyllene** | 1,26 | 1.00 | 0.80 | 1.07 | 0.86 |
| 1446 | ***α*-humulene** | 0.16 | 0.12 | 0.10 | - | - |
| 1471 | ***trans*-muurola-3,5-diene** | - | - | - | 0.09 | - |
| 1472 | **geranyl propanoate** | - | - | - | 0.12 | - |
| 1505 | ***β*-bisabolene** | 1.19 | 0.92 | - | - | - |
| 1507 | ***γ*-cadinene** | - | - | - | 0.18 | - |
| 1518 | ***δ-*amorphene** | 0.14 |  |  |  |  |
| 1552 | **thymohydro quinone** | 0.13 |  |  |  |  |
| 1569 | **spathulenol** | 0.11 |  |  |  |  |
| 1573 | **caryophyllene oxide** | 0.34 | 0.69 | 0.43 | 1.33 | - |
| **Total %** |  | 99.98 | 99.82 | 98.57 | 99.14 | 98.37 |

**Table S3.** % yield (w/w) of extracts after ultrasound assisted extraction deriving from oregano and thyme superior and inferior plant material.

| **Plant species** | **Plant material** | | **Code** | **%yield (w/w)** |
| --- | --- | --- | --- | --- |
|  | | ***Hydroalcoholic extracts*** | | |
| Oregano | Superior (grade A) | | **ORV1_WM** | 30.6% |
|  | Superior (grade B) | | **ORV2_WM** | 19.3% |
|  | Inferior (grade W) | | **ORVW_WM** | 4.10% |
| Thyme | Superior (grade A) | | **THV_WM** | 28.0% |
|  | Inferior (grade W) | | **THVW_WM** | 18.6% |

**Table S4.** Total phenolic content and DPPH free scavenging capacity of hydroalcoholic extracts by USE.

| **Plant species** | **Plant material** | **Code** | **% DPPH inhibition** | | | **TPC** |
| --- | --- | --- | --- | --- | --- | --- |
|  |  |  | **200 μg/mL** | **100 μg/mL** | **50 μg/mL** | **mg GAE/g dry weight** |
| ***Hydroalcoholic extracts*** | | | | | | |
| Oregano | Superior (grade A) | **ORV1_WM** | 91.5 ± 0.1 | 87.1 ± 0.2 | 60.3 ± 2.6 | 160.1 ± 8.0 |
|  | Superior (grade B) | **ORV2_WM** | 79.2 ± 1.7 | 78.1 ± 1.0 | 53.8 ± 2.1 | 151.3 ± 6.5 |
|  | Inferior (grade W) | **ORVW_WM** | 78.1 ± 1.0 | 78.1 ± 1.0 | 52.5 ± 1.5 | 143.8 ± 7.0 |
| Thyme | Superior (grade A) | **THV_WM** | 85.9 ± 0.6 | 66.6 ± 0.8 | 28.6 ± 0.8 | 177.2 ± 8.2 |
|  | Inferior (grade W) | **THVW_WM** | 82.7 ± 0.7 | 78.6 ± 2.7 | 42.8 ± 2.6 | 166.4 ± 4.7 |

**Table S5** GC-MS analysis of cyclohexane extracts of by-products after MAE of all plant material (superior and inferior plant) contained the main olive oil components. Prior to analysis, samples were pre-processed (derivatization) in order to transform the fatty acids into their respective volatile methyl esters.

| **RT (MIN)** | **Constituents** | **%** |
| --- | --- | --- |
| 31.18 | 9- Hexadecenoic acid, methyl ester, (z)- | 0.77 |
| 31.65 | Hexadecenoic acid, methyl ester | 12.88 |
| 34.78 | 9,12- Octadecenoic acid (z,z)-, methyl ester, (z) | 6.57 |
| 35.01 | 9- Octadecenoic acid, methyl ester, (E-) | 63.72 |
| 35.37 | Methyl stereate | 2.31 |
| **Total %** |  | **86.25** |

**FIGURES**

**GC-MS Chromatograms of Essential Oils**


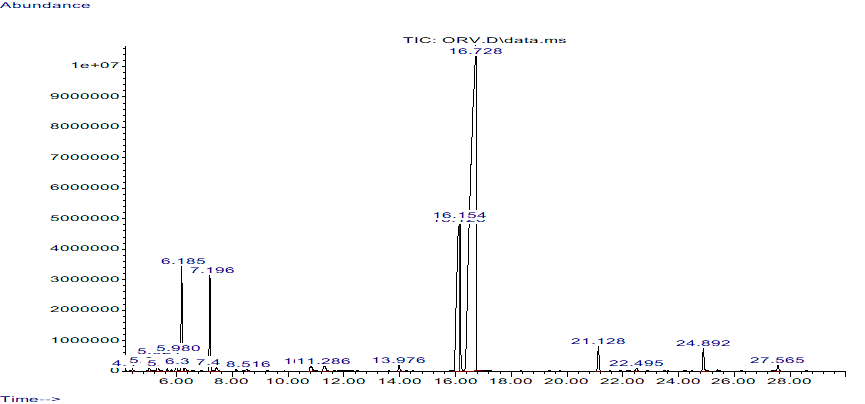


Figure S1: GC - MS chromatogram of grade A oregano essential oil (ORV1).


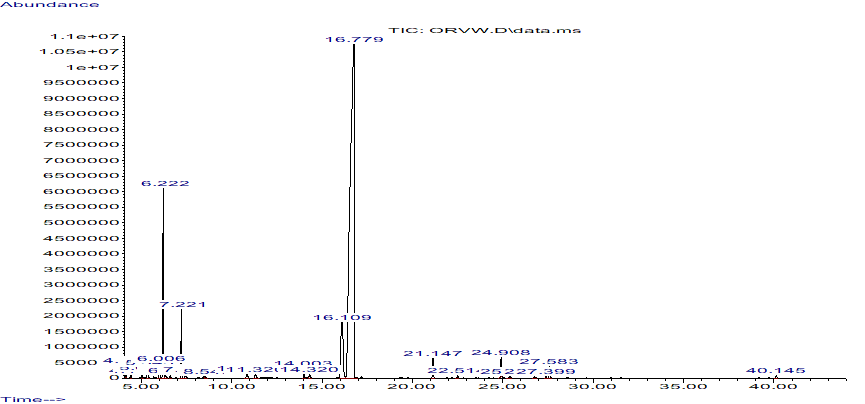


Figure S2: GC - MS chromatogram of grade B oregano essential oil (ORV2).


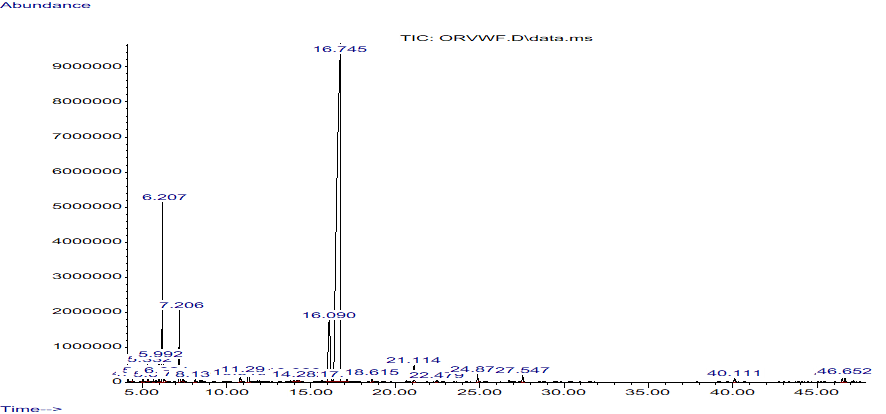


Figure S3: GC - MS chromatogram of oregano grade W essential oil (ORVW).

Figure S4: GC - MS chromatogram of grade A thyme essential oil (THV)

Figure S5: GC - MS chromatogram of thyme grade W essential oil (THVW).

**HS-SPME-GC-MS Chromatograms of olive oil and aromatic olive oils**

Figure S6: HS-SPME-GC - MS chromatogram of extra virgin olive oil used for extraction.


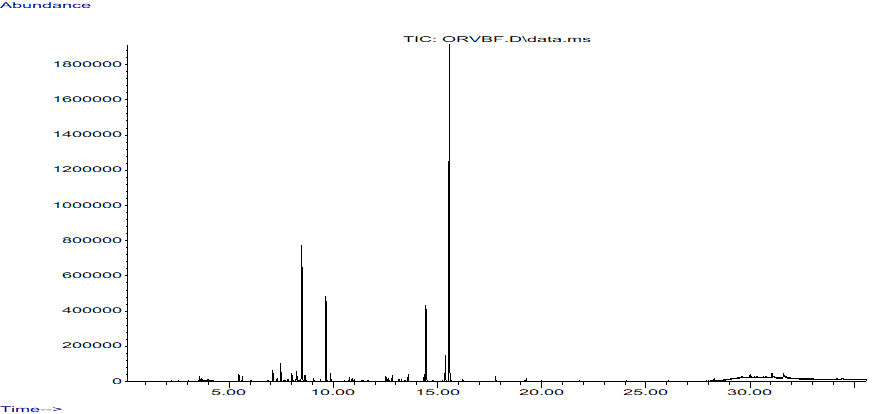


Figure S7: HS-SPME-GC - MS chromatogram of oregano grade A aromatic olive oil (ORV1_AOO).

Figure S8: HS-SPME-GC - MS chromatogram of oregano grade B aromatic olive (ORV2_AOO).

Figure S9: HS-SPME-GC - MS chromatogram of oregano grade W aromatic olive.


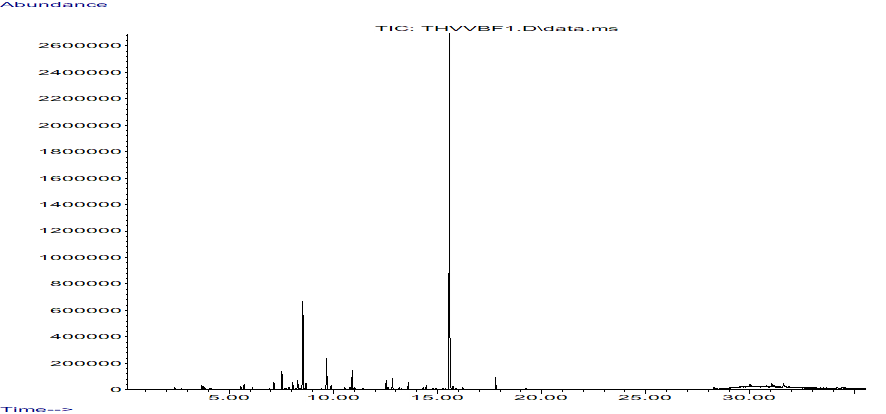


Figure S10: HS-SPME-GC - MS chromatogram of thyme grade A aromatic olive oil (THV_AOO).

Figure S11: HS-SPME-GC - MS chromatogram of thyme grade W aromatic olive oil.

**GC-MS Chromatograms of cyclohexane extracts after derivatisation**

Figure S12: GC - MS chromatogram of cyclohexane extract after derivatization of extra virgin olive oil used for extraction.

Figure S13: GC - MS chromatogram of cyclohexane extract after derivatization of oregano grade A plant material.

Figure S14: GC - MS chromatogram of cyclohexane extract after derivatization of oregano grade B plant material.

Figure S15: GC - MS chromatogram of cyclohexane extract after derivatization of oregano grade W plant material.

Figure S16: GC - MS chromatogram of cyclohexane extract after derivatization of thyme grade A plant material.

Figure S17: GC - MS chromatogram of cyclohexane extract after derivatization of thyme grade W plant material.
